# Burden of multidrug and extensively drug-resistant ESKAPEE pathogens in a secondary hospital care setting in Greece

**DOI:** 10.1017/S0950268822001492

**Published:** 2022-09-23

**Authors:** Evangelos I. Kritsotakis, Dimitra Lagoutari, Efstratios Michailellis, Ioannis Georgakakis, Achilleas Gikas

**Affiliations:** 1 Laboratory of Biostatistics, School of Medicine, University of Crete, Crete, Greece; 2 Biopathology and Microbiology Laboratory, General Hospital of Agios Nikolaos, Crete, Greece; 3 Internal Medicine Department, General Hospital of Agios Nikolaos, Crete, Greece; 4 Section of Internal Medicine, School of Medicine, University of Crete, Crete, Greece

**Keywords:** ESKAPE, hospital epidemiology, microbial drug resistance, mortality, secondary care, survival analysis

## Abstract

Bacterial antibiotic resistance (AMR) is a significant threat to public health, with the sentinel ‘ESKAPEE’ pathogens, being of particular concern. A cohort study spanning 5.5 years (2016–2021) was conducted at a provincial general hospital in Crete, Greece, to describe the epidemiology of ESKAPEE-associated bacteraemia regarding levels of AMR and their impact on patient outcomes. In total, 239 bloodstream isolates were examined from 226 patients (0.7% of 32 996 admissions) with a median age of 75 years, 28% of whom had severe comorbidity and 46% with prior stay in ICU. Multidrug resistance (MDR) was lowest for *Pseudomonas aeruginosa* (30%) and *Escherichia coli* (33%), and highest among *Acinetobacter baumannii* (97%); the latter included 8 (22%) with extensive drug-resistance (XDR), half of which were resistant to all antibiotics tested. MDR bacteraemia was more likely to be healthcare-associated than community-onset (RR 1.67, 95% CI 1.04–2.65). Inpatient mortality was 22%, 35% and 63% for non-MDR, MDR and XDR episodes, respectively (*P* = 0.004). Competing risks survival analysis revealed increasing mortality linked to longer hospitalisation with increasing AMR levels, as well as differential pathogen-specific effects. *A. baumannii* bacteraemia was the most fatal (14-day death hazard ratio 3.39, 95% CI 1.74–6.63). Differences in microbiology, AMR profile and associated mortality compared to national and international data emphasise the importance of similar investigations of local epidemiology.

## Introduction

Antibiotic-resistant infections, particularly those caused by multidrug-resistant (MDR) organisms in healthcare settings, pose a serious threat to global public health. In 2019, antimicrobial resistance (AMR) was estimated to have directly caused 1.27 million (95% CI 0.91–1.71) deaths worldwide [1] which reflects the serious impact of AMR on the efficiency of antibiotic therapy [2], and the lack of new compounds active against such organisms, specifically, *Enterococcus faecium*, *Staphylococcus aureus*, *Klebsiella pneumoniae*, *Acinetobacter baumannii*, *Pseudomonas aerugin*osa and *Enterobacter species* – (ESKAPE) [3]. The latter group was highlighted as being of critical or high priority by the World Health Organization [4] which called for urgent research and antibiotic development for these and other pathogens. ESKAPE organisms together with *Escherichia coli* (hereafter referred to as ESKAPEE) were responsible for more than 80% of global deaths attributable to AMR organisms in 2019 [1].

Greece is among the European countries with a high prevalence of antibiotic-resistant ESKAPEE infections in acute care hospitals and an estimated annual incidence of > 25 000 patients with nosocomial infections caused by difficult-to-treat pathogens [5]. Although several national and international investigations have made important contributions on the burden of AMR, they are less informative of local situations where the highest priority pathogens may differ in various locations and settings [1, 6]. Additionally, existing national AMR surveillance programmes are mainly based on phenotypic data from clinical routine without detailed information on patient characteristics, and consequently may over-represent high-risk patients in tertiary care hospitals [7]. In contrast, surveillance is minimal, and data are sparse from provincial hospitals providing secondary level care to local communities and small geographical regions.

Examining bloodstream isolates is important for monitoring AMR and its disease burden, because bacteraemia is almost universally associated with clinical infection, whereas recovery of pathogenic organisms from non-sterile sites is less likely to represent true infection [8]. Moreover, there is an ongoing important discussion on the use of hospital-onset bacteraemia as an outcome measure reflecting quality of care, and potentially a publicly reported quality metric [9, 10].

The purpose of this study was to estimate the incidence of ESKAPEE-associated bacteraemia, characterise affected patients, examine AMR patterns and trends, and assess their impact on mortality and length of hospital stay (LOS) in a setting typical of public secondary care provision in Greece.

## Methods

### Study design

This retrospective cohort study was conducted at the General Hospital of Agios Nikolaos in Crete, Greece, based on all consecutive admissions from July 2016 to December 2021 reporting positive blood cultures and antimicrobial susceptibility results in the database of the hospital's microbiology laboratory. Included were all patients who had a blood culture isolate belonging to one of the seven species of the ESKAPEE group. The number of eligible patients over the study period determined the study size and no *a priori* statistical calculation of sample size was performed.

### Setting

The study site is a 140-bed city hospital that serves a population of *ca*. 75 500 citizens and admits *ca*. 6000 patients annually (average LOS of 4 days, all-cause inpatient mortality of 3%). The hospital offers highly differentiated acute-care services in 11 medical and surgical specialities and takes referrals from other district hospitals and primary care centres in the island of Crete and nearby islands. A dedicated three-member committee comprising a clinical microbiologist, an infectious disease physician and a dedicated nurse oversee infection surveillance and control activities in the hospital.

### Data collection

For each patient, we recorded age, sex, ICD-10 codes for conditions present on admission, history of hospitalisation in the previous year, dates of admission and discharge, department of admission, department at time of blood culture, date of culture, microorganism(s) isolated, antibiotic susceptibility test results and patient outcome (classified as discharge alive, transfer to another hospital or inpatient death). In repeat confirmatory cultures, only the first blood culture results were recorded. Severity of comorbid conditions was measured by the Charlson comorbidity index (CCI), which was calculated based on the ICD-10 codes of present-on-admission comorbidities using methods previously described [11].

### Microbiology

Blood cultures were processed using the BACTEC 9050 automated instrument (Becton Dickinson, Barelas SA, Greece). Isolates were identified to species level by phenotypic tests and antimicrobial susceptibility was determined with the MicroScan autoSCAN-4 System (Beckman Coulter, Leriva SA, Greece). *E* tests were used for susceptibility testing of relevant species isolates to colistin, acinetobacter to tigecycline (breakpoint 0.55 mg/l), and staphylococci and enterococci to vancomycin (breakpoint 4 mg/l).

### Definitions

The following terms were defined prior to data analysis. Bacteraemia was classified as *healthcare-associated* if onset occurred on or after the third hospital day [10, 11], or earlier in patients receiving haemodialysis, and/or, admitted in a healthcare facility in the previous 90 days; otherwise, it was considered as *community-onset*. Onset of bacteraemia was defined as the date of collection of the blood sample for culture and polymicrobial bacteraemia was classed as isolation of two or more species isolates from the same blood culture.

Isolates with intermediate antibiotic susceptibility, or resistance, were grouped together to form the *non-susceptible* group. Each isolate was classified as follows: (i) non-MDR if non-susceptible to up to two antimicrobial groups; (ii) MDR if non-susceptible to three or more, but susceptible to at least two groups; (iii) extensively drug-resistant (XDR) if susceptible to only one or two groups; and (iv) pandrug-resistant (PDR) if non-susceptible to all agents in all antimicrobial groups. An isolate was reported as non-susceptible to an antimicrobial group if it was non-susceptible to at least one agent within the group. Antimicrobial groups and agents were defined according to the lists compiled by the ECDC and the US Centers for Disease Control and Prevention [12]. As not all required antimicrobials were routinely tested (see Supplementary Table S1), the PDR category was interpreted as ‘possible’ in this study.

### Statistical analysis

Multivariable Poisson regression with log-link function and robust variance estimation [13] was utilised to estimate adjusted relative risks (aRR) and 95% CI values for factors associated with the MDR/XDR phenotypes compared with non-MDR isolates. Investigated factors included patient sex, age, CCI, ICU stay, pre-culture LOS, healthcare-associated episode, polymicrobial growth and pathogen species. Age (years) and LOS (days) were modelled as continuous covariates.

To describe crude inpatient mortality over time and compare between pathogens and their AMR levels, cumulative incidence function plots based on competing risks analysis of survival times were utilised [14]. Differential effects between early and delayed fatalities were examined by assessing deaths in 14 and 30 days after the onset of bacteraemia and upon discharge from the hospital. Survival times were administratively right censored for patients who remained hospitalised after 14 or 30 days, respectively. Censoring of survival times was assumed non-informative for patients transferred to other hospitals before end of follow-up. Discharge alive was analysed as a competing event to in-hospital death [14]. Cause-specific hazard ratios (csHR) and their CIs were estimated with the Cox proportional hazards model to describe the direct effect of bacteraemia caused by each pathogen on the two competing events of interest (i.e. discharge alive and inpatient death). A low csHR for discharge alive reflected a low daily rate of discharge resulting in a prolonged LOS. We adjusted for confounding effects by age, ICU stay and CCI as common baseline risk factors for both prolonged LOS and increased mortality.

Full antibiogram data could not be retrieved for four (1.7%) isolates recovered from three (1.3%) patients, and these were excluded from related analyses. Data management and statistical analyses were performed using Stata version 17 (Stata Corp., College Station, TX, USA); statistical significance was indicated at the *P* < 0.05 threshold.

### Ethics and reporting

The study was approved by the hospital's Review Board and the 7th Regional Health Authority of Greece (approval no. 34392) and is reported in accordance with the Strengthening the Reporting of Observational Studies in Epidemiology (STROBE) guidelines [15].

## Results

### ESKAPEE bacteraemia incidence and affected patients

Over the 5.5-year study period, the hospital admitted 32 996 patients for 131 654 patient-days (mean LOS, 4 days; 1009 inpatient deaths; crude mortality 3%). We recovered 239 ESKAPEE bloodstream isolates from 226 patients (0.7% of all admissions); the pooled rate was 1.8 cases (95% CI 1.6–2.1) per 1000 patient-days.

Affected patients had a median age of 75 years, 57% were male, 28% had a CCI ≥ 1, 67% had history of previous hospitalisation and 44% were treated in the ICU. The median LOS before onset of bacteraemia was 2 days, but most episodes (80%) were healthcare-associated. After bacteraemia onset, 14-day, 30-day and overall, in-hospital case-fatality rates were 20%, 27% and 30%, respectively (Table 1).

**Table 1.** Demographic and clinical characteristics and outcomes of patients (*n* = 226) with bacteraemia due to an ESKAPEE pathogen

Characteristic	*n* (%)^a^
Male sex	129 (57.1)
Median age (IQR), years	74.9 (64.1–82.6)
Charlson comorbidity index	
0	162 (71.7)
1	45 (19.9)
2+	19 (8.4)
Hospitalisation in prior 12 months	152 (67.3)
Department at time of blood culture	
Intensive care unit	99 (43.8)
Medical ward	88 (38.9)
Dialysis unit	21 (9.3)
Surgical ward	15 (6.6)
Paediatric or obstetrics ward	1 (0.4)
Emergency department	2 (0.9)
Median pre-culture LOS (IQR), days	2.0 (1.0–9.0)
Healthcare-associated bacteraemia	182 (80.5)
Polymicrobial blood culture	12 (5.3)
Organism	
*E. coli*	74 (32.7)
*Enterococcus* spp.^b^	44 (19.5)
*S. aureus*	42 (18.6)
*A. baumannii*	37 (16.4)
*K. pneumoniae*	21 (9.3)
*P. aeruginosa*	10 (4.4)
*Enterobacter* spp.^c^	11 (4.9)
14-day outcome^d^	
Remain hospitalised	64 (28.3)
Discharged alive	100 (44.2)
Transferred to other hospital	16 (7.1)
Died in hospital	46 (20.4)
30-day outcome^d^	
Remain hospitalised	18 (8.0)
Discharged alive	127 (56.2)
Transferred to other hospital	19 (8.4)
Died in hospital	62 (27.4)
Hospitalisation outcome	
Discharged alive	138 (61.1)
Transferred to other hospital	21 (9.3)
Died in hospital	67 (29.6)
Median post-culture LOS (IQR), days	9.0 (6.0–16.0)

IQR, interquartile range; LOS, length of hospital stay.

a Data are presented as *n* (%) for categorical variables and median (IQR) for continuous variables. The sum of reported percentages for organisms exceeds 100% due to polymicrobial infections.

b *Enterococcus* spp. includes *E. faecium* (*n* = 9), *E. faecalis* (*n* = 21) and other or not-specified enterococci (*n* = 14).

c *Enterobacter* spp. includes *E. cloacae* (*n* = 10) and *E. aerogenes* (*n* = 1).

d From the time of bacteraemia onset.

### Pathogen and AMR distributions and trends

**Fig. 1.**
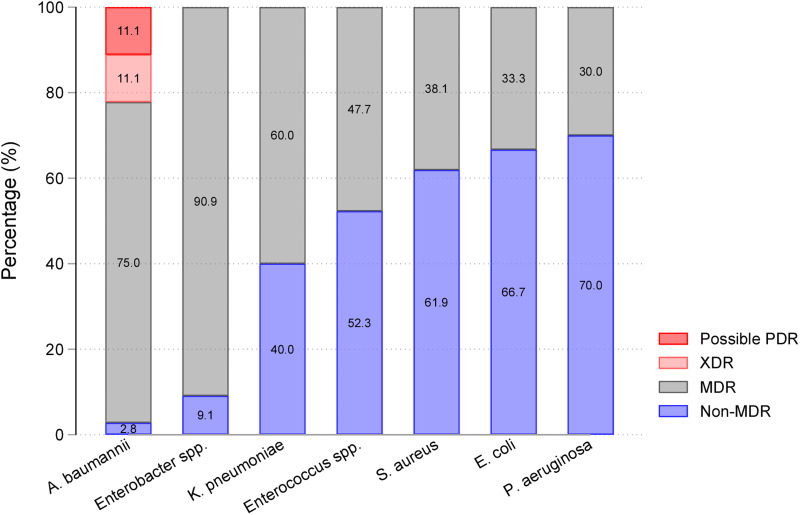
Resistance levels of ESKAPEE bloodstream isolates (*n* = 235). Four isolates were excluded as full antibiogram data were missing (one *A. baumannii*, two *E. coli* and one *K. pneumoniae*).

*E. coli* (33%) was the most frequent isolate, followed by *Enterococcus* spp. (20%), *S. aureus* (19%) and *A. baumannii* (16%). The distributions of multi-resistance levels among isolates are shown in Figure 1. Distributions of non-susceptibility rates for commonly used antibiotics per pathogen are given in Supplementary Table S2. *A. baumannii* was highly non-susceptible (>80% to 100%) to all antimicrobial agents tested, expect colistin (33%), and was almost exclusively classified as MDR or higher (97%), including four each of XDR and ‘possibly PDR’ phenotypes. None of the other pathogens were classed as XDR. MDR was most frequent in *Enterobacter* spp. (91%) and *K. pneumoniae* (60%), and less so in *E. coli* (33%) and *P. aeruginosa* (30%). A significant trend of increasing MDR incidence was observed in ESKAPEE isolates over the study period (mean annual increase of 0.1 isolates per 1000 patient-days; *P* = 0.036). *S. aureus* showed a statistically significant trend of increasing incidence of the MDR phenotype over time (Supplementary Table S3).

Risk factors for MDR or XDR (as opposed to non-MDR) ESKAPEE bacteraemia are presented in Table 2. When evaluated using a multivariable model, MDR/XDR bacteraemia was more likely to be healthcare-associated than community-onset (aRR 1.67, 95% CI 1.04–2.65). The risk of these resistance phenotypes was also significantly more associated for patients with *A. baumannii* (aRR 1.90, 95% CI 1.23–2.92) or *Enterobacter* spp. bacteraemia (aRR 1.92, 95% CI 1.26–2.94) relative to those with *E. coli*. No significant association was found between the risk of MDR/XDR bacteraemia and patient sex, age, comorbidity, pre-culture LOS or prior ICU stay.

**Table 2.** Multivariable analysis of factors associated with the MDR or XDR as opposed to non-MDR phenotypes in patients with bacteraemia (*n* = 223)

Variable	No. (%) of patients		Relative risk estimation		
	Non-MDR (*n* = 106)	MDR/XDR (*n* = 117)	aRR	95% CI	*P*
Male sex	68 (64.2%)	59 (50.4%)	0.85	0.68–1.07	0.172
Median age (IQR), years	76.2 (68.7–82.8)	72.5 (61.2–82.6)	0.99	0.99–1.00	0.124
Charlson comorbidity index					
0	70 (66.0%)	90 (76.9%)	1.00		
1	30 (28.3%)	15 (12.8%)	0.64	0.42–0.97	0.035
2+	6 (5.7%)	12 (10.3%)	1.09	0.76–1.56	0.642
Prior ICU stay	34 (32.1%)	69 (59.0%)	1.24	0.94–1.63	0.129
Median pre-culture LOS (IQR) days	1.0 (1.0–2.0)	5.0 (1.0–14.0)	1.00	0.99–1.01	0.819
Healthcare-associated bacteraemia	75 (70.8%)	104 (88.9%)	1.67	1.04–2.65	0.032
Organism^a^					
*E. coli*	44 (41.5%)	23 (19.7%)	1.00		
*A. baumannii*	1 (0.9%)	31 (26.5%)	1.90	1.23–2.92	0.004
*Enterobacter spp.*	0 (0.0%)	9 (7.7%)	1.92	1.26–2.94	0.003
*Enterococcus spp.*	18 (17.0%)	16 (13.7%)	1.15	0.70–1.90	0.587
*K. pneumoniae*	7 (6.6%)	12 (10.3%)	1.52	0.93–2.49	0.093
*P. aeruginosa*	7 (6.6%)	3 (2.6%)	0.62	0.23–1.68	0.351
*S. aureus*	25 (23.6%)	15 (12.8%)	1.03	0.60–1.76	0.909
Polymicrobial growth	4 (3.8%)	8 (6.8%)	1.31	0.74–2.33	0.356

non-MDR, non-multidrug resistant; MDR, multidrug resistant; XDR, extensively drug resistant; aRR, adjusted risk ratio; CI, confidence interval; ICU, intensive care unit; LOS, length of stay; IQR, interquartile range.

a Patients with polymicrobial cultures were assigned the highest level of resistance among the pathogens isolated.

### Clinical impact

In total, 67 of the study patients (30%) died in the hospital; these were generally older, and significantly more likely to have a CCI ≥ 2, an ICU stay prior to the onset of the bacteraemia and isolation of *A. baumannii* of an MDR or XDR phenotype (Supplementary Table S4). A trend of increasing in-hospital mortality with increasing AMR level was also evident (Supplementary Fig. S1). Case fatality rates were 22%, 35% and 63% for non-MDR, MDR and XDR episodes, respectively (*P* = 0.004 for trend).

**Fig. 2.**
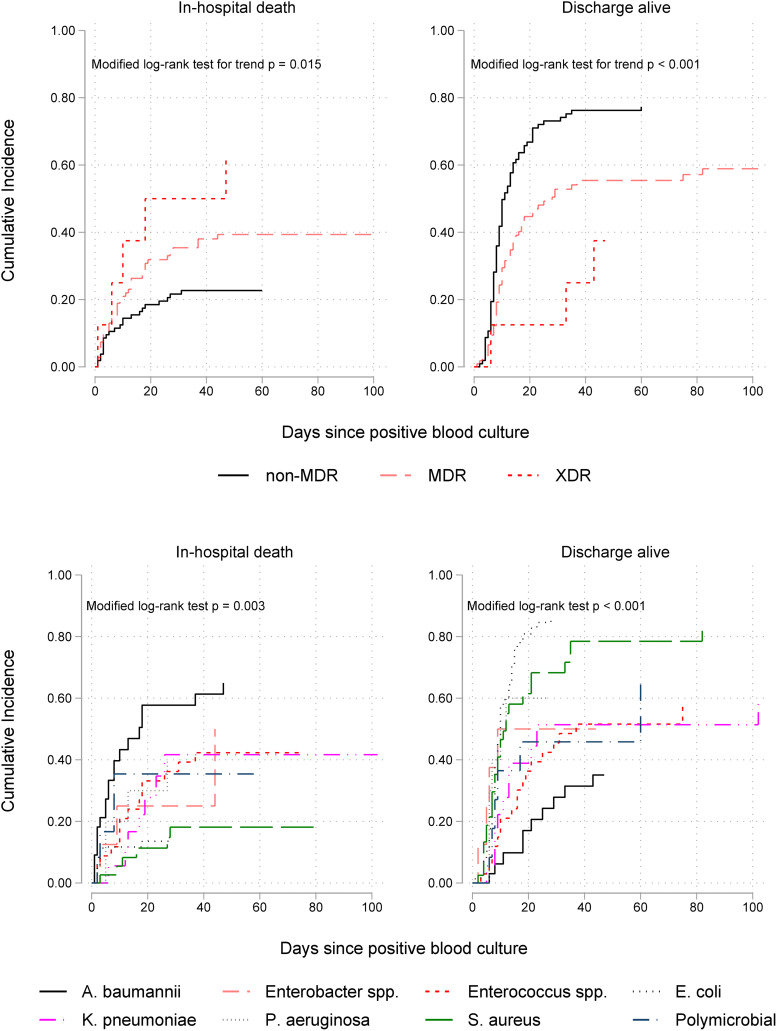
Cumulative incidence functions for in-hospital mortality (on the left) and discharge alive (on the right) by antimicrobial resistance level (upper panel) and ESKAPEE organism (lower panel) isolated from blood in 226 patients. Lower incidence of hospital discharge alive indicates longer hospitalisation after bacteraemia onset. MDR, multidrug resistant; XDR extensively drug resistant.

Cumulative incidence function plots, which account for competing risks and LOS censoring, showed that the disparity between the rates of in-hospital death in patients with non-MDR, MDR and XDR isolates was rapid in the first few days following bacteraemia onset and significantly increased with time (Fig. 2). In addition, the upper right panel in this figure shows a significant trend of decreasing discharge-alive rates, thereby increasing duration of hospital stay, with higher AMR levels. Stratification by pathogen genus (lower panels) revealed differential clinical impacts; *A. baumannii* was associated with the highest mortality rate and S. *aureus* the lowest. By contrast, *E. coli* and *S. aureus* bacteraemia had the shortest LOS (highest discharge rates) while *A. baumannii* and *Enterococcus* spp. episodes had the highest LOS.

As seen in Table 3, the multivariable competing risks analysis of 14-day outcome controlling for patient age, prior ICU stay and CCI, confirmed that of all ESKAPEE pathogens *A. baumannii* bacteraemia was the most fatal (csHR 3.39, 95% CI 1.74–6.63) with *S. aureus* the least fatal (csHR 0.27, 95% CI 0.08–0.87). Bacteraemia caused by *Enterococcus* spp. was independently associated with a prolonged LOS (csHR 0.44, 95% CI 0.23–0.85). Pathogen-specific effects on mortality and LOS diminished but remained significantly high when the analysis time was extended to 30 days after the onset of bacteraemia (Table 3) and at the time of hospital discharge (Supplementary Table S5).

**Table 3.** Multivariable competing risks survival analysis of in-hospital mortality up to 14 and 30 days after the onset of ESKAPEE-associated bacteraemia

Variable	14-day outcome						30-day outcome					
	Inpatient death			Discharge alive			Inpatient death			Discharge alive		
	csHR	95% CI	*P*	csHR	95% CI	*P*	csHR	95% CI	*P*	csHR	95% CI	*P*
Age, 10 years increase^a^	1.70	1.24–2.35	0.001	0.93	0.83–1.05	0.236	1.46	1.15–1.87	0.002	0.92	0.83–1.02	0.111
Prior ICU stay^a^	2.71	1.12–6.56	0.027	0.17	0.09–0.32	<0.001	2.12	1.00–4.53	0.051	0.16	0.09–0.28	<0.001
Charlson index (ref. = 0)^a^												
1	2.18	1.06–4.48	0.034	1.65	0.95–2.86	0.074	1.72	0.89–3.31	0.104	1.71	1.05–2.79	0.032
2+	2.16	0.89–5.23	0.089	0.35	0.08–1.49	0.158	1.98	0.94–4.17	0.073	0.36	0.11–1.17	0.089
Organism (ref. = any other)^b^												
*A. baumannii*	3.39	1.74–6.63	<0.001	0.58	0.20–1.67	0.312	2.58	1.43–4.65	0.002	0.84	0.41–1.75	0.647
*P. aeruginosa*	1.29	0.39–4.28	0.682	2.88	1.24–6.68	0.014	1.63	0.58–4.57	0.356	2.47	1.07–5.68	0.034
*K. pneumoniae*	0.56	0.17–1.82	0.331	0.84	0.41–1.74	0.643	0.93	0.42–2.09	0.867	0.75	0.39–1.43	0.380
*E. coli*	0.96	0.42–2.17	0.919	1.20	0.77–1.87	0.408	0.78	0.37–1.65	0.510	1.38	0.93–2.06	0.114
*Enterobacter* spp.	1.85	0.57–6.05	0.306	1.67	0.59–4.68	0.331	1.26	0.39–4.07	0.697	1.14	0.41–3.16	0.804
*S. aureus*	0.27	0.08–0.87	0.028	1.49	0.91–2.45	0.116	0.42	0.18–0.98	0.044	1.34	0.86–2.09	0.196
*Enterococcus* spp.	0.78	0.39–1.58	0.493	0.44	0.23–0.85	0.015	0.74	0.40–1.37	0.339	0.49	0.29–0.82	0.007
Resistance level (ref. = non-MDR)^c^												
MDR	1.32	0.68–2.57	0.406	0.80	0.52–1.23	0.318	1.15	0.64–2.04	0.641	0.92	0.62–1.35	0.658
XDR	1.61	0.45–5.78	0.467	0.62	0.08–4.67	0.638	1.19	0.39–3.66	0.756	0.31	0.04–2.32	0.254

csHR, cause-specific hazard ratio; CI, confidence interval; *P*, *P*-value; ICU, intensive care unit; MDR, multi-drug resistance; XDR, extensive drug resistance.

a The fitted model includes age, ICU stay, Charlson comorbidity index and organism.

b Each organism was entered separately into the model as a binary indicator variable to estimate its effect compared to any other organism and adjusting for age, ICU stay and Charlson index.

c Due to high correlation between resistance level and organism, a separate model was fitted to estimate the effect of resistance level adjusting for age, ICU stay and Charlson index.

Regarding the independent prognostic effects of multi-resistance patterns, our results were consistent with an increased risk of 14-day death associated with the presence of MDR (csHR 1.32, 95% CI 0.68–2.57) and XDR (csHR 1.61, 95% CI 0.45–5.78) phenotypes, independently of the patient's underlying condition, but these associations were not statistically significant.

## Discussion

In this study, bacteraemia due to sentinel ESKAPEE pathogens occurred in about seven of every 1000 hospitalisations. The episodes were primarily healthcare-associated and affected patients were characterised by advanced age, severe comorbidity and/or underlying illness requiring intensive care, previous exposure to healthcare facilities and high inpatient mortality. An increasing trend of the MDR phenotype was evident over time, mostly related to healthcare-associated bacteraemia due to *A. baumannii* or *Enterobacter* spp. An increasing level of AMR was associated not only with increasing mortality, but also with simultaneously lengthier LOS. This is important because prolonged hospitalisation increases healthcare costs, raises the risk of acquiring other nosocomial infections, and the spread of AMR, to other vulnerable patients. In addition, our analysis demonstrated a differential clinical impact depending on the pathogen. Bacteraemia caused by *A. baumannii* (almost exclusively MDR, including some XDR strains) was the most fatal while S. *aureus* infection was the least fatal. Moreover, *Enterococcus* spp. bacteraemia was independently associated with prolonged LOS.

Directly comparing the observed incidence and associated mortality of bacteraemia in this study to other studies is problematic. Most research has focused on the burden of hospital-onset (or nosocomial) bloodstream infections and a systematic review reported incidence rates per 1000 patient-days of 0.5 in France, 0.6 in England, 0.8 in Finland, 1.3 in Spain and 0.8 in the USA [16]. Our study included only sentinel ESKAPEE bacteria, but based on results of the multinational SENTRY surveillance programme, these organisms constituted at least 70% of all bloodstream infections [17]. Considering that 80% of our patient cohort were healthcare-associated, the observed rate of 1.8 cases per 1000 patient-days is largely comparable to the aforementioned studies and probably lies at the upper end of the range of reported rates in other countries. Inpatient mortality observed here (20% at 14 days, 30% overall) is within the spectrum of case fatality for bloodstream infections typically reported from studies in Europe (12–32%) and the USA (15–30%) [16]. However, given the observed differential epidemiology within the ESKAPEE group it is more appropriate to discuss their health burden in a pathogen-specific context.

In many studies, *E. coli* and *S. aureus* are numerically the most frequent, and with *P. aeruginosa*, constitute the most prevalent reported organisms causing mortality [16, 18]. *P. aeruginosa*, *K. pneumoniae* and staphylococci are typically associated with healthcare acquisition, while *E. coli* is more frequent among community-acquired cases [18]. Our data are consistent with these observations. Nevertheless, the unique burden of *A. baumannii* found in this study is noteworthy and possibly reflects its greater incidence in the Greek hospital system as a whole, both in terms of its hospital-wide frequency and detrimental impact on patient outcome [5].

According to the results of SENTRY [17], *A. baumannii* first appeared in the worldwide top-10 list of pathogens causing hospital-onset bacteraemia after 2005. It is more frequent in Europe than North America, and even more prevalent in the Asia-Pacific and Latin America regions [17]. Once considered of low virulence, the species is now recognised as a significant healthcare pathogen [19]. In Greece, a national-level study found *A. baumannii* to be the second most prevalent pathogen causing various hospital-acquired infections in 37 hospitals in 2012, and attributed more than 10 000 inpatient deaths annually in the country to carbapenem-resistant *A. baumannii* infections [5]. In this study, *A. baumannii* was an important cause of bacteraemia (>16% of cases) and the leading cause of inpatient mortality. Reasons behind the widespread persistence of *A. baumannii* in Greek hospitals have not been fully investigated, but it is likely related to the intrinsic resistance of the organism to many antibiotics and its abilities to survive in inanimate environments for long periods along with rapid development of new resistances, and clonal spread. Moreover, a robust global seasonal (temperature-associated) pattern of *Acinetobacter* infections in hospitalised patients has been recognised [20], and thereby environmental or climatic factors may impact on the local epidemiology of this organism.

AMR in *Acinetobacter* spp. is a widespread problem in Europe where combined resistance to fluoroquinolones, aminoglycosides and carbapenems was noted for more than half of invasive isolates reported to EARS-Net from 29 countries in 2020 (Europe-wide mean 34.1%) [7]. In this study, *A. baumannii* bloodstream isolates were highly non-susceptible (>80% to 100%) to all agents tested except colistin, and about one in five isolates were XDR including four strains classified as possible PDR. One-third of A. *baumannii* isolates were *colistin* non-susceptible and this proportion reached 50% during 2020 and 2021, constituting an alarming finding for our region.

We noted a significant trend of increasing MDR *S. aureus* (24% MRSA) over time, but associated bacteraemia was the least fatal outcome for our patients compared to other pathogens. We should note, however, that *S. aureus* bloodstream infection continues to carry a serious prognosis, as inpatient mortality after acquisition of the organism was 7.5% and 15% at 14 and 30 days, respectively, which is in line with large multinational cohorts at tertiary care centres reporting corresponding rates of 11–15% and 21–22%, respectively (including post-discharge deaths) [21, 22]. Similar to our results, MRSA was not independently associated with survival in those studies [22]. Likewise, no adverse association between MRSA and patient survival was reported in a large cohort of Australian patients with *S. aureus* bacteraemia after adjustment for important prognostic factors [23]. This observation that mortality was irrespective of methicillin resistance status perhaps advances a case towards novel interventions such as decolonisation strategies [24].

In general, AMR levels in this study were similarly high as those reported in the Mediterranean, including Italy and Israel [7, 18]. However, except for *A. baumannii*, none of the other pathogens presented an XDR phenotype. Major changes and challenges in the epidemiology of *E. coli* and *K. pneumoniae* bloodstream infections in Europe have been the emergence of resistance to fluoroquinolones (FQ) and third-generation cephalosporins (3GC), the latter primarily caused by extended spectrum *β*-lactamase enzymes [7, 18]. We observed resistance rates in *E. coli* and *K. pneumoniae* in our region that were much lower than the national databases reported for Greece, and closer to (but higher than) the European averages. In particular, FQ resistance of *E. coli* and *K. pneumoniae* in this study reached 25% and 45%, respectively, as compared to 24% and 34% Europe-wide and 33% and 74% Greece-wide in 2020, respectively [7]. Similarly, *E. coli* and *K. pneumoniae* 3GC resistance rates were 12% and 45%, compared respectively to Europe as a whole (15% and 34%) and wider Greece (22% and 75%) [7]. Our overall proportion of difficult-to-treat carbapenem-resistant *K. pneumoniae* was 25%, which is above the European average (10%) but well below the national average (66%) [7]. These relatively low AMR levels may partially explain the absence of independent adverse associations of *E. coli* and *K. pneumoniae* bacteraemia with inpatient mortality observed here after adjustment for indices of the patients' underlying condition.

Our findings should be interpreted in view of several limitations. First, although our local setting is typical of public secondary care provision in Greece, there may be substantial variations within the country such as clinical practice patterns, patient characteristics and microbiological investigation across hospitals in different regions. Second, we reported in-hospital mortality, which possibly underestimates the true case fatality rate, as some patients may die at home or at post-acute-care facilities soon after hospital discharge. Third, despite controlling for key prognostic indices of patients' underlying condition, residual confounding factors cannot be wholly ruled out. We could not account for treatment factors in our analysis and assessing whether worse clinical outcomes were due to intrinsic virulence properties of the offending strains or due to treatment failure, were beyond the scope of this study. Fourth, we were unable to disentangle interactions between AMR level and pathogen genus due to the high correlation between them (e.g. *A. baumannii* was exclusively MDR/XDR) and because the small sample size per pathogen did not permit pathogen-specific subgroup analyses. Finally, genetic, or molecular testing to examine specific resistance genes or enzymes was not performed; hence, the origin, clonal relationship and nosocomial spread of ESKAPEE isolates were not investigated.

In conclusion, this first comprehensive epidemiological report of bacteraemia caused by sentinel ESKAPEE pathogens in a setting typical of secondary care provision in Greece shows that it remains a rare event for hospitalised patients in this setting, but the overall incidence may lie in the higher end of the range reported from other studies in Europe. Our analysis revealed pathogen-specific differences in incidence, AMR levels, case fatality and prolongation of LOS, and identified the unique role of MDR *A. baumannii* as a leading cause of inpatient mortality. Moreover, our AMR data showed some marked deviations from national and international benchmarks, and further emphasises the importance of local investigations in healthcare epidemiology.

## Data Availability

All relevant data of this study are available from the corresponding author, EIK, upon reasonable request and subject to full anonymisation of patient data.

## Supplementary material

For supplementary material accompanying this paper visit http://doi.org/10.1017/S0950268822001492. click here to view supplementary material
