# Supplementary material for: Burden of multidrug and extensively drug-resistant ESKAPEE pathogens in a secondary hospital care setting in Greece
Source: Epidemiol Infect. 2022 Sep 23;150:e170. doi: 10.1017/S0950268822001492 (PMC9981128; doi:10.1017/S0950268822001492)
Supplement: Supplementary file 1 [file hygsup.zip › S0950268822001492sup002.docx]

Epidemiology and Infection

**Burden of multidrug and extensively drug-resistant ESKAPEE pathogens in a secondary hospital care setting in Greece**

Evangelos I. Kritsotakis*^1^, Dimitra Lagoutari^1^, Efstratios Michailellis^2^, Ioannis Georgakakis^3^, and Achilleas Gikas^4^.

*^1^ Laboratory of Biostatistics, School of Medicine, University of Crete, Crete, Greece.*

*^2^ Biopathology Laboratory, General Hospital of Agios Nikolaos, Crete, Greece.*

*^3^ Internal Medicine Department, General Hospital of Agios Nikolaos, Crete, Greece.*

*^4^ Section of Internal Medicine, School of Medicine, University of Crete, Crete, Greece.*

**Supplementary Material**

Table of Contents

[Table S1. Antimicrobial agents available and tested in each antimicrobial category used to define resistance levels (MDR, XDR, PDR) for each ESKAPEE pathogen 2](#_Toc113717717)

[Table S2. Overall and annual non-susceptibility proportions for commonly used antimicrobials of ESKAPEE-E bloodstream isolates (n = 235), July 2016 - December 2021. 4](#_Toc113717718)

[Table S3. Time trends of incidence rates of ESKAPEE organisms (n = 235) isolated in blood cultures, July 2016 - December 2021. 7](#_Toc113717719)

[Figure S1. In-hospital mortality according to antimicrobial resistance levels of ESKAPEE bloodstream isolates in 236 patients. 9](#_Toc113717720)

[Table S4. Univariate analysis of overall in-hospital mortality following the onset of ESKAPEE bacteraemia 10](#_Toc113717721)

[Table S5. Multivariable competing risks survival analysis of overall in-hospital mortality and discharge alive after the onset of ESKAPEE bacteraemia. 11](#_Toc113717722)

## Table S1. Antimicrobial agents available and tested in each antimicrobial category used to define resistance levels (MDR, XDR, PDR) for each ESKAPEE pathogen

| **Antimicrobial category** | **Antimicrobial agent** | ***S. aureus*** | ***Enterococcus* spp.** | ***E. coli,***  ***K. pneumonia,***  ***Enterobacter* spp.** | ***P. aeruginosa*** | ***A. baumannii*** |
| --- | --- | --- | --- | --- | --- | --- |
| Aminoglycosides | Gentamicin | T | T | - | T | T |
|  | Gentamycin high level | - | - | T | - | - |
|  | Tobramycin | - | - | T | T | T |
|  | Amikacin | - | - | T | T | T |
|  | Netilmicin | - | - | NT | NT | NT |
|  | Streptomycin (high level) | - | T | - | - | - |
| Ansamycins | Rifampin/rifampicin | NT | - | - | - | - |
| Anti-MRSA cephalosporins | Ceftaroline | T | - | NT | - | - |
| Anti-staphylococcal b-lactams | Oxacillin | T | - | - | - | - |
| Antipseudomonal penicillins  + b-lactamase inhibitors | Ticarcillin-clavulanic acid | - | - | NT | NT | NT |
|  | Piperacillin-tazobactam | - | - | T | T | T |
| Carbapenems | Ertapenem | - | - | T | - | - |
|  | Imipenem | - | NT | T | T | T |
|  | Meropenem | - | NT | T | T | T |
|  | Doripenem | - | NT | NT | NT | NT |
| Cephalosporins, 1^st^ and 2^nd^ generation | Cefazolin | - | - | NT | - | - |
|  | Cefuroxime | - | - | T | - | - |
| Cephalosporins, 3^rd^ and 4^th^ generation | Cefotaxime | - | - | T | - | T |
|  | Ceftriaxone | - | - | - | - | NT |
|  | Ceftazidime | - | - | T | T | T |
|  | Cefepime | - | - | T | T | T |
| Cephamycins | Cefoxitin | T | - | T | - | - |
|  | Cefotetan | - | - | NT | - | - |
| Fluoroquinolones | Ciprofloxacin | T | T | T | T | T |
|  | Levofloxacin | - | T | - | T | T |
|  | Moxifloxacin | NT | NT | - | - | - |
| Folate pathway inhibitors | Trimethoprim - sulphamethoxazole | T | - | T | - | T |
| Fucidanes | Fusidic acid | NT | - | - | - | - |
| Glycopeptides | Vancomycin | T | T | - | - | - |
|  | Teicoplanin | T | T | - | - | - |
|  | Telavancin | NT | NT | - | - | - |
| Glycylcyclines | Tigecycline | NT | NT | T | - | T |
| Lincosamides | Clindamycin | T | - | - | - | - |
| Lipopeptides | Daptomycin | T | T | - | - | - |
| Macrolides | Erythromycin | T | - | - | - | - |
| Monobactams | Aztreonam | - | - | NT | NT | - |
| Oxazolidinones | Linezolid | T | T | - | - | - |
| Penicillins | Ampicillin | - | T | T | - | - |
| Penicillins + b-lactamase inhibitors | Amoxicillin-clavulanic acid | - | - | NT | - | - |
|  | Ampicillin-sulbactam | - | - | T | - | T |
| Phenicols | Chloramphenicol | NT | - | NT | - | - |
| Phosphonic acids | Fosfomycin | T | - | T | T | - |
| Polymyxins | Colistin | - | - | T | T | T |
|  | Polymyxin B | - | - | - | NT | NT |
| Streptogramins | Quinupristin - dalfopristin | NT | NT | - | - | - |
| Tetracyclines | Tetracycline | T | T | T | - | T |
|  | Doxycycline | NT | NT | NT | - | NT |
|  | Minocycline | NT | NT | NT | - | NT |
| Proportion of groups with ≥ 1 agent tested: | | 12/17 | 8/10 | 14/17 | 7/8 | 9/9 |
| Proportion of agents tested: | | 14/23 | 10/19 | 20/31 | 12/17 | 16/23 |

**Abbreviations.** T, tested; NT, not tested; - , not applicable.

**Note.** Criteria for defining MDR: non-susceptible to ≥1 agent in ≥ 3 antimicrobial categories; XDR: non-susceptible to ≥1 agent in all but ≤2 categories; PDR: non-susceptible to all antimicrobial agents listed as applicable.

## Table S2. Overall and annual non-susceptibility proportions for commonly used antimicrobials of ESKAPEE-E bloodstream isolates (n = 235), July 2016 - December 2021.

| **Microorganism** | **Antimicrobial(s)** | **Per cent non-susceptible (%)** | | | | | | | **P value for linear trend ^a^** |
| --- | --- | --- | --- | --- | --- | --- | --- | --- | --- |
|  |  | **Total** | **2016** | **2017** | **2018** | **2019** | **2020** | **2021** |  |
| *A. baumannii* (n=36) | AMK GEN TOB | 97.2 | 100.0 | 100.0 | 100.0 | 100.0 | 100.0 | 87.5 | . |
|  | IMP MEM | 97.2 | 100.0 | 100.0 | 100.0 | 100.0 | 100.0 | 87.5 | . |
|  | CIP LVX | 97.2 | 100.0 | 100.0 | 100.0 | 100.0 | 100.0 | 87.5 | . |
|  | TZP | 100.0 | 100.0 | . | . | . | 100.0 | . | . |
|  | CAZ CTX FEP | 97.2 | 100.0 | 100.0 | 100.0 | 100.0 | 100.0 | 87.5 | . |
|  | SXT | 83.3 | 66.7 | 57.1 | 100.0 | 66.7 | 100.0 | 100.0 | 0.037 |
|  | SAM | 97.2 | 100.0 | 100.0 | 100.0 | 100.0 | 100.0 | 87.5 | . |
|  | CST | 33.3 | 0.0 | 42.9 | 33.3 | 0.0 | 57.1 | 42.9 | 0.137 |
|  | TET | 90.6 | 100.0 | 100.0 | 100.0 | 100.0 | 85.7 | 75.0 | 0.171 |
|  | TGC | 85.7 | 100.0 | 100.0 | 100.0 | 100.0 | 50.0 | 71.4 | 0.165 |
| *P. aeruginosa* (n=10) | AMK GEN TOB | 30.0 | . | . | 40.0 | 0.0 | 50.0 | 0.0 | 0.647 |
|  | IMP MEM | 20.0 | . | . | 20.0 | 0.0 | 0.0 | 100.0 | 0.386 |
|  | CAZ FEP | 40.0 | . | . | 60.0 | 0.0 | 0.0 | 100.0 | 0.712 |
|  | CIP LVX | 10.0 | . | . | 20.0 | 0.0 | 0.0 | 0.0 | . |
|  | TZP | 50.0 | . | . | 60.0 | 50.0 | 0.0 | 100.0 | 0.763 |
|  | FOF | 37.5 | . | . | 25.0 | 50.0 | 100.0 | 0.0 | 0.796 |
|  | CST | 40.0 | . | . | 50.0 | 0.0 | 0.0 | 100.0 | 0.643 |
| *K. pneumoniae* (n=20) | AMK GEN TOB | 31.6 | 100.0 | 25.0 | 25.0 | 0.0 | 50.0 | 25.0 | 0.314 |
|  | TZP | 35.0 | 100.0 | 25.0 | 20.0 | 33.3 | 50.0 | 25.0 | 0.418 |
|  | IMP MEM ETP | 25.0 | 100.0 | 25.0 | 20.0 | 0.0 | 50.0 | 0.0 | 0.101 |
|  | CXM | 50.0 | 100.0 | 50.0 | 20.0 | 66.7 | 100.0 | 25.0 | 0.495 |
|  | CAZ CTX FEP | 45.0 | 100.0 | 25.0 | 20.0 | 66.7 | 100.0 | 25.0 | 0.793 |
|  | FOX | 55.6 | 100.0 | 50.0 | 33.3 | 66.7 | 100.0 | 25.0 | 0.392 |
|  | CIP | 45.0 | 100.0 | 50.0 | 20.0 | 33.3 | 50.0 | 50.0 | 0.592 |
|  | SXT | 40.0 | 100.0 | 25.0 | 40.0 | 0.0 | 100.0 | 25.0 | 0.504 |
|  | TGC | 11.8 | 0.0 | 25.0 | 0.0 | 0.0 | 50.0 | 0.0 | 0.979 |
|  | SAM | 57.9 | 100.0 | 50.0 | 40.0 | 50.0 | 100.0 | 50.0 | 0.826 |
|  | FOF | 29.4 | 0.0 | 25.0 | 33.3 | 100.0 | 0.0 | 25.0 | 0.748 |
|  | TET | 17.6 | 0.0 | 25.0 | 0.0 | 0.0 | 100.0 | 0.0 | 0.656 |
|  | CST | 33.3 | 0.0 | 0.0 | 100.0 | . | . | . | . |
| *E. coli* (n=72) | AMK GEN TOB | 11.4 | 25.0 | 10.0 | 0.0 | 12.5 | 16.7 | 10.0 | 0.824 |
|  | TZP | 10.3 | 14.3 | 10.5 | 23.1 | 0.0 | 0.0 | 10.0 | 0.359 |
|  | IMP MEM ETP | 1.4 | 0.0 | 0.0 | 7.1 | 0.0 | 0.0 | 0.0 | 0.823 |
|  | CXM | 25.4 | 25.0 | 15.8 | 42.9 | 12.5 | 41.7 | 10.0 | 0.979 |
|  | CAZ CTX FEP | 12.5 | 12.5 | 15.0 | 0.0 | 12.5 | 33.3 | 0.0 | 0.869 |
|  | FOX | 0.0 | 0.0 | 0.0 | 0.0 | 0.0 | 0.0 | 0.0 | . |
|  | CIP | 24.6 | 16.7 | 25.0 | 21.4 | 12.5 | 41.7 | 22.2 | 0.553 |
|  | SXT | 17.4 | 0.0 | 21.1 | 25.0 | 25.0 | 25.0 | 0.0 | 0.893 |
|  | TGC | 0.0 | 0.0 | 0.0 | 0.0 | 0.0 | 0.0 | 0.0 | . |
|  | SAM | 30.6 | 25.0 | 30.0 | 21.4 | 50.0 | 25.0 | 40.0 | 0.522 |
|  | FOF | 0.0 | 0.0 | 0.0 | 0.0 | 0.0 | 0.0 | 0.0 | . |
|  | TET | 40.0 | 0.0 | 75.0 | 0.0 | 50.0 | 40.0 | 0.0 | 0.364 |
|  | CST | 0.0 | . | 0.0 | . | . | . | . | . |
| *Enterobacter spp.* (n=11) | AMK GEN TOB | 18.2 | 0.0 | 0.0 | 0.0 | 50.0 | 0.0 | 25.0 | 0.468 |
|  | TZP | 36.4 | 0.0 | 0.0 | 0.0 | 50.0 | 0.0 | 75.0 | 0.100 |
|  | IMP MEM ETP | 63.6 | 100.0 | 0.0 | 0.0 | 50.0 | 100.0 | 100.0 | 0.122 |
|  | CXM | 81.8 | 100.0 | 0.0 | 100.0 | 100.0 | 0.0 | 100.0 | 0.538 |
|  | CAZ CTX FEP | 54.5 | 0.0 | 0.0 | 50.0 | 50.0 | 0.0 | 100.0 | 0.078 |
|  | FOX | 100.0 | 100.0 | 100.0 | 100.0 | 100.0 | 100.0 | 100.0 | . |
|  | CIP | 9.1 | 0.0 | 0.0 | 0.0 | 50.0 | 0.0 | 0.0 | 0.911 |
|  | SXT | 36.4 | 0.0 | 0.0 | 0.0 | 50.0 | 0.0 | 75.0 | 0.100 |
|  | TGC | 0.0 | 0.0 | 0.0 | 0.0 | 0.0 | 0.0 | 0.0 | . |
|  | SAM | 54.5 | 100.0 | 0.0 | 0.0 | 50.0 | 100.0 | 75.0 | 0.315 |
|  | FOF | 27.3 | 0.0 | 0.0 | 50.0 | 0.0 | 100.0 | 25.0 | 0.567 |
|  | TET | 9.1 | 0.0 | 0.0 | 0.0 | 50.0 | 0.0 | 0.0 | 0.911 |
| *Enterococcus spp.* (n=44) | GEN | 37.5 | 50.0 | 33.3 | 80.0 | 57.1 | 20.0 | 10.0 | 0.047 |
|  | STR | 48.3 | 0.0 | 100.0 | 100.0 | 50.0 | 20.0 | 33.3 | 0.147 |
|  | CIP LVX | 72.1 | 75.0 | 50.0 | 80.0 | 57.1 | 83.3 | 90.9 | 0.105 |
|  | TEC VAN | 9.1 | 0.0 | 0.0 | 16.7 | 14.3 | 0.0 | 18.2 | 0.254 |
|  | DAP | 0.0 | 0.0 | 0.0 | 0.0 | 0.0 | 0.0 | 0.0 | . |
|  | LZD | 48.4 | 0.0 | 0.0 | 25.0 | 28.6 | 100.0 | 70.0 | 0.007 |
|  | AMP | 22.7 | 25.0 | 20.0 | 33.3 | 42.9 | 0.0 | 18.2 | 0.569 |
|  | TET | 60.5 | 50.0 | 77.8 | 50.0 | 71.4 | 33.3 | 63.6 | 0.694 |
| *S. aureus* (n=42) | GEN | 5.6 | 0.0 | 0.0 | 0.0 | 33.3 | 0.0 | 10.0 | 0.517 |
|  | CPT | 8.7 | 0.0 | 0.0 | 0.0 | 0.0 | 20.0 | 14.3 | 0.312 |
|  | FOX OXA | 23.8 | 0.0 | 20.0 | 0.0 | 33.3 | 9.1 | 60.0 | 0.053 |
|  | CIP | 36.7 | 0.0 | 33.3 | 20.0 | 33.3 | 20.0 | 75.0 | 0.044 |
|  | SXT | 4.9 | 0.0 | 0.0 | 0.0 | 33.3 | 9.1 | 0.0 | 0.660 |
|  | TEC VAN | 16.7 | 0.0 | 0.0 | 40.0 | 33.3 | 9.1 | 30.0 | 0.198 |
|  | CLI | 35.9 | 0.0 | 22.2 | 25.0 | 66.7 | 27.3 | 66.7 | 0.045 |
|  | DAP | 0.0 | 0.0 | 0.0 | 0.0 | 0.0 | 0.0 | 0.0 | . |
|  | ERY | 53.7 | 66.7 | 20.0 | 40.0 | 100.0 | 54.5 | 77.8 | 0.055 |
|  | LZD | 45.8 | 0.0 | 0.0 | 66.7 | 66.7 | 40.0 | 62.5 | 0.100 |
|  | FOF | 0.0 | 0.0 | 0.0 | 0.0 | 0.0 | 0.0 | 0.0 | . |
|  | TET | 10.0 | 33.3 | 0.0 | 0.0 | 33.3 | 20.0 | 0.0 | 0.806 |

**Abbreviations.** AMK: Amikacin, SAM: Ampicillin-Sulbactam, AMP: Ampicillin, FEP: Cefepime, CTX: Cefotaxime, FOX: Cefoxitin, CPT: Ceftaroline, CAZ: Ceftazidime, CXM: Cefuroxime, CIP: Ciprofloxacin, CLI: Clindamycin, CST: Colistin, DAP: Daptomycin, ETP: Ertapenem, ERY: Erythromycin, FOF: Fosfomycin, GEN: Gentamicin, IPM: Imipenem, LVX: Levofloxacin, LZD: Linezolid, MEM: Meropenem, MXF: Moxifloxacin, OXA: Oxacillin, TZP: Piperacillin-Tazobactam, STR: Streptomycin high level, TEC: Teicoplanin, TET: Tetracycline, TGC: Tigecycline, TOB: Tobramycin, SXT: Trimethoprim-sulphamethoxazole, VAN: Vancomycin

**Notes:**

**^a^** P-value calculated using logistic regression to assess the statistical significance of an increasing or decreasing linear trend in annual non-susceptibility proportions.

## Table S3. Time trends of incidence rates of ESKAPEE organisms (n = 235) isolated in blood cultures, July 2016 - December 2021.

| **Pathogen** | **Resistance level** | **Annual incidence rate per 1,000 patient-days** | | | | | | | **Annual trend statistics ^a^** | |
| --- | --- | --- | --- | --- | --- | --- | --- | --- | --- | --- |
|  |  | **Total** | **2016** | **2017** | **2018** | **2019** | **2020** | **2021** | **Trend ^b^ (95%CI)** | ***P*** |
| *A.baumannii* | Non-MDR | 0.01 | 0.00 | 0.00 | 0.00 | 0.00 | 0.00 | 0.05 | 0.01 (-0.00 - 0.02) | 0.110 |
|  | MDR | 0.21 | 0.49 | 0.18 | 0.11 | 0.12 | 0.26 | 0.24 | -0.01 (-0.06 - 0.04) | 0.740 |
|  | XDR | 0.06 | 0.00 | 0.07 | 0.04 | 0.00 | 0.15 | 0.09 | 0.02 (-0.01 - 0.06) | 0.169 |
|  | Total | 0.28 | 0.57 | 0.26 | 0.15 | 0.12 | 0.41 | 0.38 | 0.02 (-0.05 - 0.09) | 0.612 |
| *P. aeruginosa* | Non-MDR | 0.05 | 0.00 | 0.00 | 0.11 | 0.08 | 0.10 | 0.00 | 0.00 (-0.02 - 0.03) | 0.820 |
|  | MDR | 0.02 | 0.00 | 0.00 | 0.08 | 0.00 | 0.00 | 0.05 | 0.00 (-0.01 - 0.02) | 0.650 |
|  | XDR | 0.00 | 0.00 | 0.00 | 0.00 | 0.00 | 0.00 | 0.00 | 0.00 . | . |
|  | Total | 0.08 | 0.00 | 0.00 | 0.19 | 0.08 | 0.10 | 0.05 | 0.01 (-0.02 - 0.04) | 0.670 |
| *K. pneumoniae* | Non-MDR | 0.06 | 0.00 | 0.07 | 0.15 | 0.00 | 0.00 | 0.09 | 0.00 (-0.02 - 0.03) | 0.908 |
|  | MDR | 0.09 | 0.16 | 0.07 | 0.04 | 0.12 | 0.10 | 0.09 | 0.00 (-0.04 - 0.04) | 0.891 |
|  | XDR | 0.00 | 0.00 | 0.00 | 0.00 | 0.00 | 0.00 | 0.00 | 0.00 . | . |
|  | Total | 0.16 | 0.24 | 0.15 | 0.19 | 0.12 | 0.10 | 0.19 | -0.00 (-0.05 - 0.04) | 0.893 |
| *E. coli* | Non-MDR | 0.36 | 0.49 | 0.51 | 0.34 | 0.16 | 0.36 | 0.38 | -0.02 (-0.09 - 0.04) | 0.493 |
|  | MDR | 0.18 | 0.16 | 0.22 | 0.19 | 0.16 | 0.26 | 0.09 | -0.02 (-0.06 - 0.03) | 0.503 |
|  | XDR | 0.00 | 0.00 | 0.00 | 0.00 | 0.00 | 0.00 | 0.00 | 0.00 . | . |
|  | Total | 0.56 | 0.73 | 0.77 | 0.53 | 0.32 | 0.61 | 0.47 | -0.05 (-0.14 - 0.04) | 0.251 |
| *Enterrobacter.spp* | Non-MDR | 0.01 | 0.00 | 0.04 | 0.00 | 0.00 | 0.00 | 0.00 | -0.01 (-0.01 - 0.00) | 0.198 |
|  | MDR | 0.08 | 0.08 | 0.00 | 0.08 | 0.08 | 0.05 | 0.19 | 0.03 (-0.00 - 0.06) | 0.094 |
|  | XDR | 0.00 | 0.00 | 0.00 | 0.00 | 0.00 | 0.00 | 0.00 | 0.00 . | . |
|  | Total | 0.08 | 0.08 | 0.04 | 0.08 | 0.08 | 0.05 | 0.19 | 0.02 (-0.01 - 0.06) | 0.190 |
| *Enterococcus.spp* | Non-MDR | 0.17 | 0.24 | 0.26 | 0.04 | 0.12 | 0.26 | 0.19 | 0.00 (-0.04 - 0.05) | 0.926 |
|  | MDR | 0.16 | 0.08 | 0.11 | 0.19 | 0.16 | 0.05 | 0.33 | 0.03 (-0.01 - 0.08) | 0.130 |
|  | XDR | 0.00 | 0.00 | 0.00 | 0.00 | 0.00 | 0.00 | 0.00 | 0.00 . | . |
|  | Total | 0.33 | 0.33 | 0.37 | 0.23 | 0.28 | 0.31 | 0.52 | 0.04 (-0.02 - 0.09) | 0.191 |
| *S. aureus* | Non-MDR | 0.20 | 0.24 | 0.29 | 0.11 | 0.04 | 0.41 | 0.14 | -0.01 (-0.07 - 0.05) | 0.803 |
|  | MDR | 0.12 | 0.00 | 0.07 | 0.08 | 0.08 | 0.15 | 0.33 | 0.06 (0.02 - 0.10) | 0.002 |
|  | XDR | 0.00 | 0.00 | 0.00 | 0.00 | 0.00 | 0.00 | 0.00 | 0.00 . | . |
|  | Total | 0.32 | 0.24 | 0.37 | 0.19 | 0.12 | 0.56 | 0.47 | 0.05 (-0.02 - 0.13) | 0.142 |
| All ESKAPEE | Non-MDR | 0.87 | 0.98 | 1.17 | 0.76 | 0.40 | 1.13 | 0.85 | -0.02 (-0.13 - 0.09) | 0.713 |
|  | MDR | 0.86 | 0.98 | 0.66 | 0.76 | 0.72 | 0.87 | 1.33 | 0.10 (0.01 - 0.20) | 0.036 |
|  | XDR | 0.06 | 0.00 | 0.07 | 0.04 | 0.00 | 0.15 | 0.09 | 0.02 (-0.01 - 0.06) | 0.169 |
|  | Total | 1.78 | 1.95 | 1.91 | 1.56 | 1.11 | 2.15 | 2.28 | 0.11 (-0.04 - 0.26) | 0.138 |

**Abbreviations.** non-MDR: non-multidrug resistant, MDR: multidrug resistant, XDR: extensively drug resistant

**Notes:**

**^a^** Estimated using ordinary least-squares linear regression of time on monthly incidence rates. Two pairs of sine-cosine Fourier functions of time were included to capture seasonality.

**^b^** The trend is the estimated annual average change in the incidence rate of bacteraemia.

## Figure S1. In-hospital mortality according to antimicrobial resistance levels of ESKAPEE bloodstream isolates in 236 patients.

## Table S4. Univariate analysis of overall in-hospital mortality following the onset of ESKAPEE bacteraemia

|  | **Discharged alive** | **Died in hospital** | ***P*** |
| --- | --- | --- | --- |
|  | **N=159** | **N=67** |  |
| Sex |  |  | 0.34 |
| Female | 65 (40.9) | 32 (47.8) |  |
| Male | 94 (59.1) | 35 (52.2) |  |
| Median age (IQR), years | 72.6 (61.5-82.6) | 78.1 (71.2-83.7) | 0.013 |
| ICU stay |  |  | <0.001 |
| No | 108 (67.9) | 14 (20.9) |  |
| Yes | 51 (32.1) | 53 (79.1) |  |
| Charlson comorbidity index |  |  | <0.001 |
| 0 | 122 (76.7) | 40 (59.7) |  |
| 1 | 31 (19.5) | 14 (20.9) |  |
| 2+ | 6 (3.8) | 13 (19.4) |  |
| Hospitalization in prior 12 months | 104 (65.4) | 48 (71.6) | 0.36 |
| Healthcare-associated bacteremia | 125 (78.6) | 57 (85.1) | 0.26 |
| Organism |  |  | <0.001 |
| *A. baumannii* | 13 (8.2) | 20 (29.9) |  |
| *Enterobacter spp.* | 6 (3.8) | 3 (4.5) |  |
| *Enterococcus spp.* | 20 (12.6) | 14 (20.9) |  |
| *E. coli* | 60 (37.7) | 9 (13.4) |  |
| *K. pneumoniae* | 12 (7.5) | 7 (10.4) |  |
| *P. aeruginosa* | 6 (3.8) | 4 (6.0) |  |
| *S. aureus* | 34 (21.4) | 6 (9.0) |  |
| Polymicrobial growth | 8 (5.0) | 4 (6.0) |  |
| Antibiotic resistance level |  |  | 0.014 |
| non-MDR | 83 (52.2) | 23 (34.3) |  |
| MDR or XDR | 74 (46.5) | 43 (64.2) |  |
| Missing | 2 (1.3) | 1 (1.5) |  |

**Abbreviations.** IQR, interquartile range; ICU, intensive care unit; non-MDR: non-multidrug resistant, MDR: multidrug resistant, XDR: extensively drug resistant.

## Table S5. Multivariable competing risks survival analysis of overall in-hospital mortality and discharge alive after the onset of ESKAPEE bacteraemia.

| **Variable** | Analysis of cause-specific hazards | | | | | |
| --- | --- | --- | --- | --- | --- | --- |
|  | **In-hospital death** | | | **Discharge alive** | | |
|  | **csHR** | **95%CI** | ***P*** | **csHR** | **95%CI** | ***P*** |
| Age, 10 years increase ^a^ | 1.54 | 1.21 - 1.96 | <0.001 | 0.92 | 0.83 - 1.01 | 0.090 |
| Prior ICU stay ^a^ | 2.16 | 1.01 - 4.60 | 0.046 | 0.16 | 0.09 - 0.28 | <0.001 |
| Charlson index (ref. = 0) ^a^ |  |  |  |  |  |  |
| 1 | 1.63 | 0.85 - 3.13 | 0.144 | 1.68 | 1.04 - 2.69 | 0.032 |
| 2+ | 2.20 | 1.08 - 4.49 | 0.030 | 0.44 | 0.16 - 1.21 | 0.112 |
| Organism (ref. = any other) ^b^ |  |  |  |  |  |  |
| *A. baumannii* | 2.60 | 1.47 - 4.59 | 0.001 | 0.91 | 0.47 - 1.77 | 0.782 |
| *P. aeruginosa* | 1.59 | 0.57 - 4.47 | 0.379 | 2.48 | 1.08 - 5.71 | 0.033 |
| *K. pneumoniae* | 0.85 | 0.38 - 1.90 | 0.684 | 0.65 | 0.35 - 1.23 | 0.189 |
| *E. coli* | 0.71 | 0.34 - 1.48 | 0.356 | 1.36 | 0.93 - 2.00 | 0.117 |
| *Enterobacter spp.* | 1.39 | 0.49 - 3.91 | 0.533 | 0.94 | 0.34 - 2.61 | 0.911 |
| *S. aureus* | 0.40 | 0.17 - 0.93 | 0.032 | 1.41 | 0.93 - 2.14 | 0.106 |
| *Enterococcus spp.* | 0.73 | 0.41 - 1.32 | 0.302 | 0.52 | 0.32 - 0.85 | 0.010 |
| Resistance level (ref. = non-MDR) ^c^ |  |  |  |  |  |  |
| MDR | 1.10 | 0.63 - 1.95 | 0.729 | 0.84 | 0.57 - 1.23 | 0.365 |
| XDR | 1.15 | 0.41 - 3.27 | 0.792 | 0.63 | 0.18 - 2.14 | 0.457 |

**Abbreviations.** csHR, cause-specific hazard ratio; CI, confidence interval; P, p-value; ICU, intensive care unit; MDR, multi-drug resistance; XDR, extensive drug resistance.

**Notes.**

^a^ The fitted model includes age, ICU stay, Charlson comorbidity index and organism.

^b^ Each organism was entered separately into the model as a binary indicator variable to estimate its effect compared to any other organism and adjusting for age, ICU stay, and Charlson index.

^c^ Due to high correlation between resistance level and organism, a separate model was fitted to estimate the effect of resistance level adjusting for age, ICU stay, and Charlson index.
